# Cerebral blood flow dynamics during cardiac surgery in infants

**DOI:** 10.1038/s41390-024-03161-z

**Published:** 2024-04-03

**Authors:** Martin Leth-Olsen, Gaute Døhlen, Hans Torp, Siri Ann Nyrnes

**Affiliations:** 1 Department of Circulation and Medical Imaging (ISB), Faculty of Medicine and Health Sciences, NTNU—Norwegian University of Science and Technology, Trondheim, Norway; 2 Children’s Clinic, St Olav’s University Hospital, Trondheim, Norway; 3 Department of Pediatric Cardiology, Oslo University Hospital, Oslo, Norway

## Abstract

**Background:**

In this pilot study, we investigated continuous cerebral blood flow velocity measurements to explore cerebrovascular hemodynamics in infants with congenital heart disease undergoing cardiac surgery.

**Methods:**

A non-invasive transfontanellar cerebral Doppler monitor (NeoDoppler) was used to monitor 15 infants (aged eight days to nine months) during cardiac surgery with cardiopulmonary bypass. Numerical and visual analyses were conducted to assess trends and events in Doppler measurements together with standard monitoring equipment. The mean flow index, calculated as the moving Pearson correlation between mean arterial pressure and time averaged velocity, was utilized to evaluate dynamic autoregulation. Two levels of impaired autoregulation were defined (Mean flow index >0.3/0.45), and percentage of time above these limits were calculated.

**Results:**

High quality recordings were achieved during 90.6% of the monitoring period. There was a significant reduction in time averaged velocity in all periods of cardiopulmonary bypass. All patients showed a high percentage of time with impaired dynamic autoregulation, with Mean flow index >0.3 and 0.45: 73.71% ± 9.06% and 65.16% ± 11.27% respectively. Additionally, the system promptly detected hemodynamic events.

**Conclusion:**

Continuous transfontanellar cerebral Doppler monitoring could become an additional tool in enhancing cerebral monitoring in infants during cardiac surgery.

**Impact:**

- This pilot study demonstrates the feasibility of continuous transfontanellar Doppler monitoring of cerebral blood flow velocities during cardiac surgery in infants.
- It also demonstrates a high proportion of time with impaired cerebral autoregulation during cardiac surgery based on the Mean flow index.
- Continuous transfontanellar Doppler could become a useful tool to improve cerebral monitoring and provide new pathophysiological insight.

## Introduction

Neurodevelopmental impairment in children with congenital heart disease (CHD) is common.^1^ Most at risk are infants requiring cardiac surgery with cardiopulmonary bypass (CPB).^2^ A recent paper showed declining prevalence of postoperative white matter injury and suggested that lower rates of hypotension improved cerebral perfusion.^3^ While maintaining optimal cerebral perfusion is a goal during CPB, monitoring cerebrovascular perfusion is challenging. Near-infrared spectroscopy (NIRS) is often used during surgery and provides information on regional cerebral tissue oxygen saturation (rScO2) and correlates with superior vena cava and jugular vein oxygen saturation.^4^ However, interpretation of rScO2 is challenging as rScO2 is regional with limited penetration and can be influenced by both oxygen availability and consumption, i.e. changes in cerebral blood flow and metabolic demands.^5^ Transcranial Doppler (TCD) or serial transfontanellar ultrasound Doppler measurements can provide continuous real-time or longitudinal data on cerebral blood flow velocities (CBFV) during CPB.^6,7^ CBFV has been shown to correlate well with changes in cerebral blood flow.^8^ In neonates and infants, a transfontanellar approach can be used to monitor perioperative CBFV with ultrasound.^9,10^

For gradual changes in blood pressure (BP), cerebral blood flow is kept relatively constant. However, during rapid fluctuations, such as during surgery, cerebral blood flow demonstrates greater variability. Dynamic autoregulation is the physiological process that attenuates these rapid changes in cerebral perfusion pressure.^11^ Neonates and infants with CHD may be vulnerable to changes in cerebral perfusion pressure due to delayed brain maturation and/or altered hemodynamics.^12^ There is no gold standard to evaluate autoregulation, but the interaction between Doppler measured CBFV or NIRS and mean arterial pressure (MAP) as surrogates for cerebral blood flow and perfusion pressure can be used to describe this relationship.^13–15^

NeoDoppler is a new transfontanellar cerebral Doppler monitor for neonates and infants.^16,17^ In this study we investigated the feasibility of including cerebral Doppler in multimodal monitoring of neonates and infants undergoing cardiac surgery with CPB. Our aim was to describe cerebrovascular hemodynamic trends and events with a special focus on the relationship between invasively measured BP and cerebral Doppler data, including investigation of the percentage of time with impaired autoregulation during the full surgical course.

## Methods

### Patient selection

The study was conducted at the Department of Pediatric Cardiology, Oslo University Hospital (OUS), Oslo, Norway, in collaboration with the ultrasound group at the Department of Circulation and Medical Imaging, The Norwegian University of Science and Technology (NTNU), Trondheim, Norway. The study was approved by the Regional Committee for Medical and Health Research Ethics, REC Central (Reference 2017/314), the Norwegian directorate of Health and The Norwegian Medicines Agency (Reference 19/05458). Written informed consent was obtained from the parents of the participants.

Patients were recruited at OUS from October 2019 to April 2021 (with a recruitment pause in 2020 due to the global pandemic). Neonates and infants under one year of age who were scheduled for cardiac surgery with CPB were eligible for inclusion. We received consent from the parents of 16 children.

### Study protocol

Patients who underwent cardiac surgery were monitored with NeoDoppler. Cerebral Doppler monitoring was initiated after intubation and continued until the end of the surgery, before transfer to the thoracic intensive care unit. A customized soft hat with a probe attachment mechanism was used to attach the probe over the anterior fontanelle, as described previously.^18^ The first author was present during the entire surgery in all patients and periodically checked the Doppler signal in order to ensure that the signal was adequate. The cerebral Doppler monitor display was otherwise blinded for the clinical personnel. Doppler data were recorded in intervals with 15-30 min recordings with up to one-minute pauses to allow for data storage and to adhere to the ALARA principle (“as low as reasonably achievable”). The perioperative care was provided according to the institutional standards, the anesthesiologist’s and surgeon’s discretion.

### Equipment, image acquisition and data collection (Fig. 1)

NeoDoppler is a non-invasive ultrasound Doppler system developed by the ultrasound group at NTNU, Trondheim, Norway. The prototype system used in this study consists of a scanner (Manus EIM-A, Aurotech Ultrsaound AS, Tydal, Norway), a small ultrasound probe (Imasonic SAS, France), operating at 7.8 MHz with plane wave transmissions covering a cylindrical shape with a diameter of 10 mm and depth down to 38 mm (Supplementary Table 1).^16^ The system is connected to a PC with an in-house MATLAB (The MathWorks, Natick, MA) application and displays real-time high-frame-rate color M-mode Doppler and Doppler spectrogram simultaneously (Supplementary Video 1). Analog invasive blood pressure was sampled from the monitoring equipment (Nihon Kohden, Tokyo, Japan) synchronized, calibrated and stored with the Doppler data. NIRS data, rScO2, was sampled from INVOS 5100c and the neonatal OxyAlert NIRSensor (Medtronic, MA) in approximately seven seconds intervals. Additional data (SpO2, FiO2, EtCO2, temperature, blood gasses, medications, CPB data) from the electronic patient chart (Metavision, iMDsoft, Tel Aviv, Israel) were retrospectively collected in one-minute intervals. In addition, the first author manually registered events in a time stamped document during the surgery.

### Doppler data analysis

The inhouse MATLAB application was used for post processing and detailed analysis of the Doppler recordings. It allows for adjustment of sample volume size and depth, gain, vertical scale, horizontal sweep through a menu system. Automatic Doppler tracings with calculation of peak systolic velocity, end diastolic velocity, time averaged maximum velocity (TAV) and resistive index (RI = (Peak systolic velocity—end diastolic velocity)/peak systolic velocity) are generated based on the tracings. The Doppler signal was optimized in post processing. The sample volume was set to five mm, the depth selected for the Doppler tracings was kept constant through the entire surgical procedure for all individual patients and the gain was adjusted to allow for optimal Doppler tracing. A trend window was then generated to allow examination for the entire surgical period. The trend window displayed CBFV tracings and indices, invasive blood pressure, SpO2, FiO2, NIRS, body temperature, EtCO2, end tidal sevoflurane, end tidal isoflurane and heart rate. Blood gasses with partial pressure of carbon dioxide and hematocrit were also collected.

### Feasibility and quality evaluation

A quality metric (0–100%) was calculated for each heartbeat, based on the correlation between consecutive heartbeats. Only quality >80% was considered as valid/high quality data. During CPB, valid data was instead defined as signal to noise ratio > six dB, since waveform correlation cannot be measured accurately during continuous non pulsatile flow. Valid fraction was defined as the time with valid data in percent of the total recording time.

### Descriptive visual analysis of monitoring parameters in each patient

**Fig. 1:**
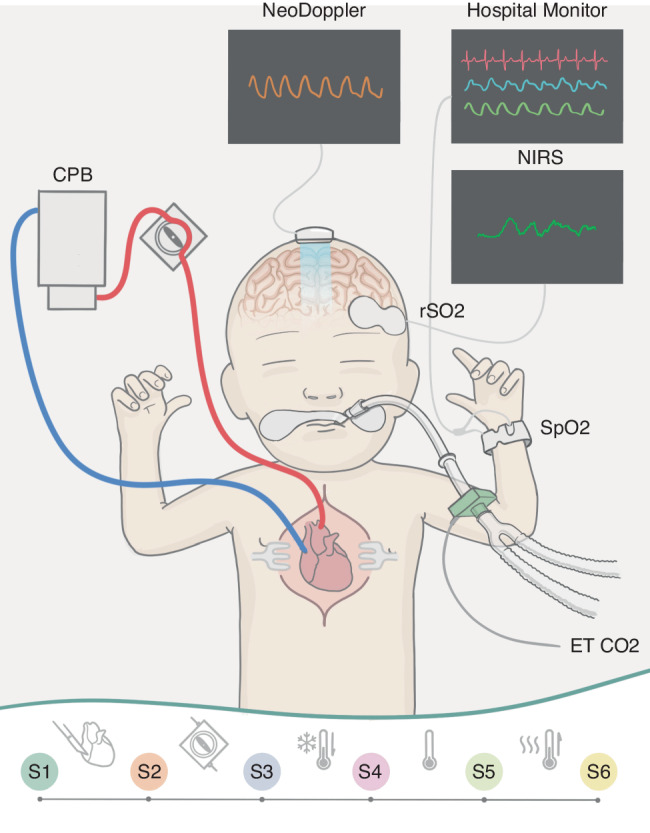
Multimodal monitoring including NeoDoppler during cardiac surgery. The figure illustrates the research setup with the NeoDoppler probe monitoring cerebral blood flow velocities through the open fontanelle during cardiac surgery with cardiopulmonary bypass. Near-infrared spectroscopy, electrocardiogram, pulse oximetry and invasive blood pressure are also monitored. The six surgical periods were defined as S1: a baseline period before start of surgery; S2: start of surgery before cardiopulmonary bypass (CPB); S3: from start of CPB, during cooling; S4: during CPB at minimal temperature; S5: during CPB while rewarming; S6: after CPB before transfer to thoracic intensive care. CPB cardiopulmonary bypass, ET CO2 end-tidal carbon dioxide, rSO2 regional oxygen saturation, SpO2 pulse oximetry oxygen saturation.

**Fig. 2:**
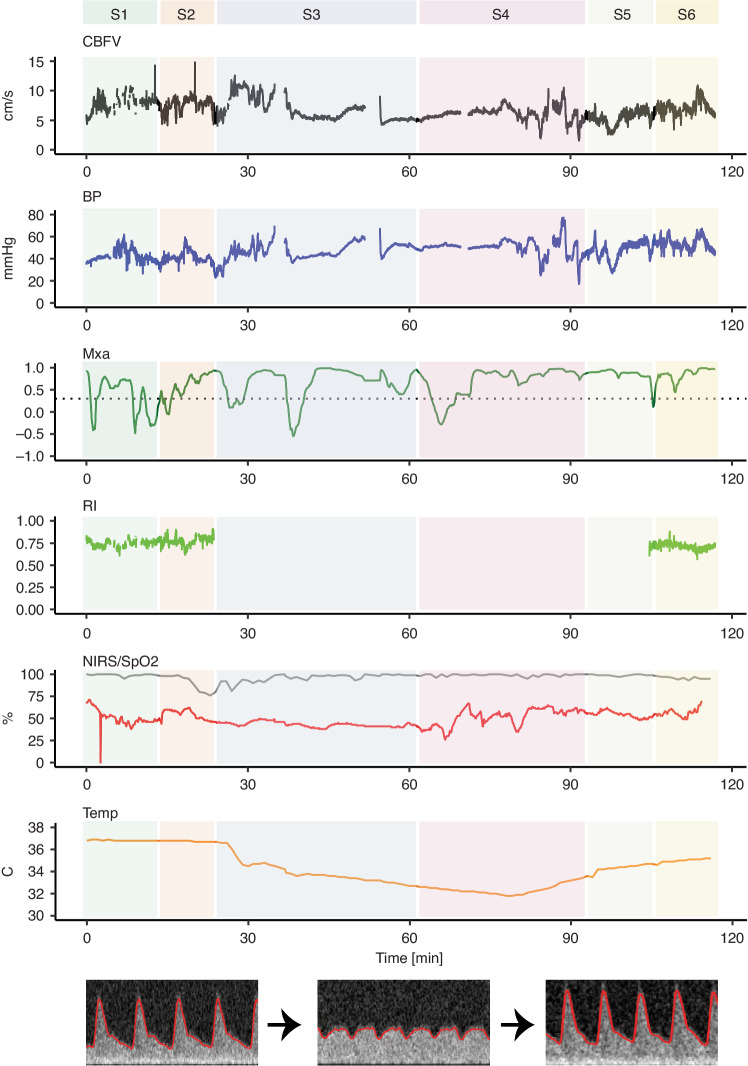
Hemodynamic trends by multimodal monitoring including NeoDoppler during cardiac surgery. An entire surgical period for one patient with cerebral blood flow velocity (CBFV) – time averaged velocity, blood pressure (BP) – mean arterial pressure, mean flow index (Mxa) with a cut off above 0.3, resistive index (RI), near-infrared spectroscopy (NIRS), oxygen saturation (SpO2) and temperature measurements illustrated. RI cannot be calculated during CPB due to the non-pulsatile flow. S1 to S6 illustrates the different periods of surgery; S1: a baseline period before start of surgery; S2: start of surgery before cardiopulmonary bypass (CPB); S3: from start of CPB, during cooling; S4: during CPB at minimal temperature; S5: during CPB while rewarming; S6: after CPB before transfer to thoracic intensive care. At the bottom of the figure, Doppler spectrograms show pre-CPB, CPB, and post-CPB flow patterns. BP blood pressure, CBFV cerebral blood flow velocity, CPB cardiopulmonary bypass, MAP mean arterial pressure, Mxa mean flow index, NIRS near-infrared spectroscopy, RI resistive index, SpO2 pulse oximetry oxygen saturation, TAV time averaged velocity, Temp temperature.

Manual visual inspection and evaluation of all Doppler recordings was performed by the first author. Artifacts and signal interference were evaluated visually. Trend windows displaying the full monitoring period, as shown in the result section (Fig. 2), were generated for all patients, and were visually evaluated by the first and last author. The monitoring data was divided in six time periods (Figs. 1, 2).

### Dynamic cerebral autoregulation

**Fig. 3:**
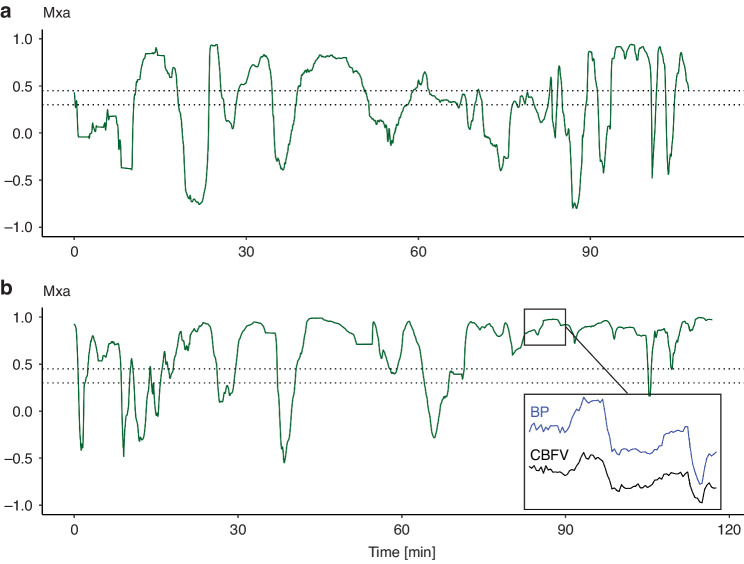
Relationship between cerebral blood flow velocity (CBFV) and blood pressure (BP). The figure illustrates the mean flow index (Mxa) for the patients with the lowest and highest percentage of time with Mxa >0.3/0.45. Panel a shows a 2.8-month-old patient with complete atrioventricular defect who had 56.9%/45.0% of the time with Mxa >0.3/0.45. Panel b shows a 3.8-month-old patient with ventricular septal defect who had 84.5%/79% of the time with Mxa >0.3/0.45. A selected period (marked with a square) of CBFV and corresponding BP measurements with high Mxa is displayed. BP blood pressure, CBFV cerebral blood flow velocity, Mxa mean flow index.

The mean flow index (Mxa) was used to evaluate dynamic autoregulation. Mxa was calculated with a custom-made Matlab script as the moving Pearson correlation coefficient between the BP and CBFV with 10 s averages calculated over 300 s for all patients, then plots were created (Fig. 3). Periods with selective cerebral circulation or deep hypothermic circulatory arrest were excluded from these analyses. Mxa ranges from minus one to one, where higher values indicate impaired autoregulation. The exact threshold of Mxa to indicate impaired autoregulation varies in studies with 0.3 most used but higher values are also reported.^19,20^ We therefore calculated percentage of time with Mxa >0.3 and >0.45 for the entire surgical course for all patients.

### Cerebral hypoxia and hypotension

Percentage of time with NIRS < 63% was calculated to describe time with cerebral hypoxia and percentage of time with mean arterial BP < -2SD for age to describe time with hypotension.^21,22^

### Monitoring trends—all patients

Common trends in the whole patient group were analyzed. The data for each patient were grouped in six different surgical periods (Fig. 1).

### Events

Major cerebrovascular events were described based on visual impressions of the trend curves and Doppler tracings.

### Statistical analysis

Statistical analysis was performed using IBM SPSS® Statistics for Windows version 28 (IBM, Armonk, NY). Categorical variables are presented as count (percent). Continuous variables are presented as mean ± standard deviation for normally distributed data. The normality assumption was assessed by visual inspection of Q-Q plots. Statistical differences between surgical periods compared to baseline were tested with paired Student’s *t* test; a *p* value < 0.05 was considered statistically significant.

### Safety

The safety of this cerebral Doppler monitoring system has previously been elaborated.^16,17^ The temperature increase is highest at the skin surface; due to the unfocused beam, the temperature diminishes with increasing depth. Thermal and mechanical indices were continuously displayed on the display unit. The skin where the probe was attached was inspected after removal to assess for local adverse effects.

## Results

### Patient characteristics

Sixteen patients were eligible for inclusion. One patient was excluded due to technical problems of the cerebral Doppler monitor during patient inclusion, leaving 15 patients (age from five days old to nine-month-old) for full analyses. Seven patients had isolated septal defects, six patients had conotruncal defects, one underwent repair for coarctation of the aorta and one had total anomalous pulmonary vein connection. All patients (100%) received isoflurane for maintenance of anesthesia (for details see Supplementary table 2). Five patients (33%) received adrenaline infusion and 4 patients (27%) received milrinone infusion at one point during surgery, with median infusion rates 0.07 mcg/kg/min and 0.5 mcg/kg/min respectively. Further data are summarized in Table 1.

**Table 1:** Basic clinical data.

Clinical data	Total (*n* = 15)
Male (n)	10 (62.5%)
Age (months)	4.16 ± 2.89
Weight (g)	5585 ± 1650.14
Length (cm)	59.54 ± 7.33
BSA (m2)	0.31 ± 0.06
PGE preop (n)	2 (13.3%)
Deep hypothermia (n)	2 (13.3%)
DHCA (n)	1 (6.7%)
MAP (mmHg)	58.87 ± 10.32
Xcorp (min)	71.6 ± 29.4
NeoDoppler monitoring period (min)	234.3 ± 73.0

Frequencies are presented as count and percentage. Continuous variables are presented as mean ± standard deviation for normally distributed data.

*BSA* body surface area, *DHCA* deep hypothermia with circulatory arrest, *PGE* prostaglandin E, *Xcorp* extracorporeal circulation.

### Feasibility and quality evaluation

All patients had good quality Doppler recordings making automated Doppler curve tracing in the spectrogram feasible. As demonstrated previously, diathermia caused artifacts in the Doppler signal and in other monitoring equipment; automatic Doppler tracings were not feasible in these periods.^17^ Manual tracing could still be possible in periods of diathermia but were not done in this study. Valid fraction as defined in the method section was calculated to 90.6% of the total monitoring time in this material.

### Trends—visual description

**Fig. 4:**
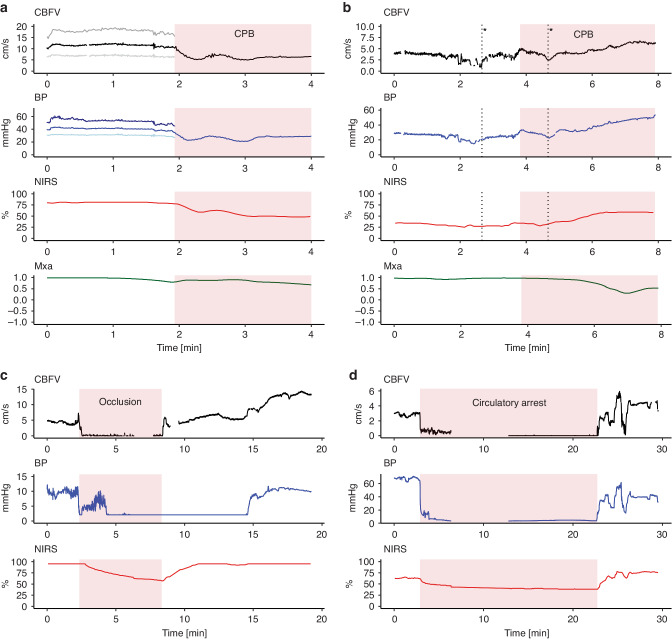
Hemodynamic events during cardiac surgery. Cerebral blood flow velocities (CBFV), blood pressure (BP) and near-infrared spectroscopy are displayed more closely during four different hemodynamic events. Corresponding mean flow index (Mxa) is displayed for panel a and b. Panel a demonstrates the transition to cardiopulmonary bypass (CPB) with a decrease in time averaged velocity (TAV), mean arterial pressure (MAP) and NIRS at the initiation of CPB in a one-month-old patient with a ventricular septal defect. Panel b demonstrates a case with severe hypotension and administration of adrenaline (*) with correlating changes in CBFV, BP and NIRS in a 3.5-month-old patient with a complete atrioventricular septal defect. Panel c demonstrates a case with accidental occlusion of the arterial cannula during selective cerebral perfusion with immediate cessation of cerebral blood flow in a six-day-old patient with coarctation of the aorta. An increase in CBFV is seen after reestablishing perfusion. A gradual decline in NIRS is also seen during occlusion. Panel d demonstrates the changes seen during deep hypothermia with circulatory arrest in a one-month-old patient with total anomalous pulmonary vein connection. BP blood pressure, CBFV cerebral blood flow velocity, CPB cardiopulmonary bypass, MAP mean arterial pressure, Mxa mean flow index, NIRS near-infrared spectroscopy, TAV time averaged velocity.

Visual inspection of trend windows revealed prominent individual differences. However, all patients had visually stable CBFV when the probe was attached before the surgical procedure started. After surgery had started, however, more rapid changes in TAV and MAP were seen without any clear visual association with temperature changes. In 13/15 patients a decrease in both TAV and MAP could be seen immediately after initiation of CPB (Fig. 4a), where eight of the 13 had a subsequently increase creating a U-shaped tracing. NIRS displayed a corresponding U-shaped pattern in five of the patients that displayed this pattern in TAV and MAP. Visually decreased TAV could been seen in ten of 15 patients during CPB compared to before CPB. TAV and MAP visually corresponded quite well during rapid changes, but slower changes during CPB did not necessarily correspond with MAP in all patients. An example of trends is illustrated in Fig. 2.

### Trends—numerical presentation of the full dataset

Basic measurements for the six different surgical periods are presented in Table 2. A reduction in TAV compared to baseline was seen in all surgical periods on CPB; S3 (t(14) = 4.644 *p* = < 0.001), S4 (t(14) = 3.725 *p* = 0.001) and S5 (t(14) = 4.793 *p* < 0.001), respectively. No statistically significant change from baseline was seen in periods S2 and S6. A significant decrease of MAP was seen in S2 (t(13) = 2.735 *p* = 0.009) and S3 (t(13) = 2.710 *p* = 0.009). A non-significant increase in MAP was seen in S4-S6. There was no significant change in RI or EtCO2 compared to baseline. NIRS showed a significant reduction from baseline in periods S2, S3 and S5 but not in S4 or S6. A significant decrease in temperature was seen in periods S2-S5. There was no significant difference in Mxa or percentage of Mxa >0.3/0.45 compared to baseline.

**Table 2:** Measurements during cardiac surgery with cardiopulmonary bypass.

	S1	S2	S3	S4	S5	S6
Time (min)	17.00 ± 7.25	12.73 ± 7.33	37.13 ± 19.58	4.85 ± 0.42	31.50 ± 16.27	23.92 ± 9.68
TAV (cm/s)	9.86 ± 4.27	9.28 ± 4.03	*6.24 ± 2.30	*6.01 ± 1.67	*6.02 ± 2.23	8.42 + - 3.83
TAV %	100	95 ± 16	69 ± 29	72 ± 40	67 ± 26	92 ± 36
MAP (mmHg)	48.84 ± 8.42	*44.99 ± 9.86	*40.27 ± 12.03	50.94 ± 12.87	50.72 ± 6.51	49.74 ± 5.52
MAP %	100	92 ± 11	83 ± 27	105 ± 26	105 ± 17	103 ± 17
MAP < -2SD	0.27% ± 0.88%	7.31% ± 16.27%	*20.96% ± 27.98%	2.49% ± 7.25%	1.03% ± 2.38%	0.15% ± 0.40%
Mxa	0.51 ± 0.34	0.65 ± 0.24	0.47 ± 0.18	0.35 ± 0.50	0.52 ± 0.22	0.58 ± 0.22
Mxa > 0.3 (%)	70.91 ± 27.67	82.06 ± 17.00	73.47 ± 15.82	66.34 ± 37.87	75.82 ± 15.34	79.31 ± 15.79
Mxa > 0.45 (%)	66.23 ± 29.81	76.15 ± 19.39	67.29 ± 16.23	57.16 ± 39.62	63.70 ± 26.31	69.93 ± 22.82
NIRS (%)	72.33 ± 15.03	*68.33 ± 15.82	*66.05 ± 14.06	65.80 ± 15.01	*63.64 ± 12.51	68.93 ± 15.20
NIRS < 63% (%)	26.24 ± 39.02	30.09 ± 40.29	38.41 ± 39.22	40.00 ± 50.71	48.34 ± 41.96	32.99 ± 41.63
Tp (C°)	36.03 ± 0.84^a^	*35.90 ± 0.86^a^	*32.38 ± 1.76	*30.66 ± 2.10	*33.44 ± 1.34	35.75 ± 0.50
EtCO2 (kPa)	5.55 ± 0.99^b^	5.75 ± 1.09	CPB			5.73 ± 1.06

Time averaged velocity (TAV), TAV %, mean arterial pressure (MAP), MAP %, Mean flow index (Mxa) and percentage of time with Mxa > 0.3, percentage of time with Mxa > 0.45, cerebral near-infrared spectroscopy (NIRS), percentage of time with NIRS < 63%, temperature (Tp) and end tidal CO2 (EtCO2) measurements presented as mean ± standard deviation at S1: a baseline period before start of surgery; S2: start of surgery before cardiopulmonary bypass (CPB); S3: from start of CPB, during cooling; S4: during CPB at minimal temperature; S5: during CPB while rewarming; S6: after CPB before transfer to thoracic intensive care.

* Significant change from baseline, *p* < 0.05, paired Student’s *t* test.

^a^Missing value for patient five and seven.

^b^Missing value for patient seven.

### Dynamic autoregulation

All patients showed more time with impaired dynamic autoregulation than time with intact autoregulation. The mean percentage of time with Mxa >0.3 per patient was 73.71% (SD ± 9.06%), Mxa >0.45 was 65.16% (SD ± 11.27%) and mean Mxa was 0.51 (SD ± 0.68). The percentage of Mxa >0.3/0.45 ranged from of 56.9%/45.0% in a patient with a complete atrioventricular septal defect to 84.5%/79.0% in a patient with ventricular septal defect. Both patients had CPB with moderate hypothermia (Fig. 3). The two patients with CPB and deep hypothermia had Mxa >0.3/0.45 of 83.2%/70.2% and 81.4%/68.1%.

### Cerebral hypoxia and hypotension

The mean percentage of time with NIRS < 63% was 37.87% (SD ± 0.33%) and the mean percentage of time with MAP < -2 SD for age was 6,64% (SD ± 7.67%).

### Hemodynamic events—examples

Four important hemodynamic events are illustrated in (Fig. 4) (a) a typical transition to CPB (Supplementary Video 1) (b) administration of adrenaline due to hypotension, (c) accidental occlusion of the arterial cannula (Supplementary Video 2) and (d) deep hypothermia with circulatory arrest. The 2 first examples illustrate the striking correlation between CBFV, BP and NIRS, while the 2 last examples illustrate the immediate loss of CBFV during circulatory arrest. One patient had accidental occlusion of the arterial cannula, where a rubber band had slipped over the arterial cannula during selective cerebral circulation and deep hypothermia. Visual inspection of the Doppler signal during postprocessing demonstrated immediate cessation of cerebral flow with TAV dropping from 3.96 m/s (SD ± 0.44) in the minute prior to occlusion to zero m/s for five minutes and 32 seconds until the surgeon noticed the rubber band and circulation was reestablished. After reperfusion, a gradual significant increase in TAV is seen to maximum 13.67 m/s (SD ± 0.37), a 345% relative increase in TAV compared to pre occlusion. A delayed response in NIRS is seen from 95% prior to occlusion before a gradual decline starts 14 seconds after occlusion, decreasing to 75% (20% reduction) after two minutes and with a nadir of 57% at the moment of reperfusion before gradually increasing to 95% over the next minutes where it stays during the period of maximum Doppler velocity measurements. As described earlier, the cerebral Doppler monitor was blinded during this period.

## Discussion

This study demonstrates that transfontanellar cerebral Doppler monitoring can provide continuous data on CBFV parameters during cardiac surgery with CPB in infants. The Doppler data shows trends and events in CBFV in a heterogenous group of patients undergoing different types of cardiac surgery with CPB. Multimodal monitoring that includes continuous cerebral Doppler adds clinically relevant information on cerebral perfusion not available with standard monitoring equipment and responds more rapidly than NIRS during hemodynamic changes.

An important finding in this study is the large proportion of time with impaired autoregulation. Optimizing blood pressure control to maintain stable cerebral perfusion in patients with CHD was highlighted in the recent paper by Peyvandi et al.^3^ A study by Votova-Smith et al. investigated cerebral autoregulation preoperatively in neonates with CHD found impaired autoregulation in 15.3% of the time and Brunsch et al. found impaired autoregulation in up to 9.3% of the time during the first 72 hours after birth in neonates with CHD.^13,23^ These studies used MAP and NIRS based correlation, in contrast to our study with Doppler-based measurements that have a substantially higher temporal resolution (424 Hz). In contrast to these studies, the children in our study were monitored during surgery, cooled and on CPB that are all factors that can potentially influence autoregulation further. It’s important to note that there is no gold standard to evaluate dynamic autoregulation and different studies use different methods that all have uncertainties and unclear clinical relevance. There are significant methodological variations in studies evaluating autoregulation with Mxa and the optimal method is still unclear.^24^ Analyzing dynamic autoregulation in the frequency domain with transfer function analysis is an alternative to Mxa. However, transfer function analysis also faces the same challenges with methodology as Mxa but standardization has been proposed.^25^ NIRS based evaluation of autoregulation is used as an alternative to Doppler based measurements. However, the correlation between these modalities is shown to be variable and NIRS measurements have challenges that also needs to be addressed.^26,27^ The exact threshold of Mxa to indicate loss of autoregulation also vary in studies.^14,19,20^ A recent study with ultrafast power Doppler during CPB in newborns with CHD also suggested significant loss of autoregulation during CPB in line with our study.^28^

Evaluating autoregulation is challenging. In his review of the literature in 1959, Lassen presented his now classic “Lassen curve”.^29^ This curve describes a plateau, i.e., a blood pressure range where autoregulation is the most effective. However, the range is better described as an area where slow changes in BP are associated with more stable cerebral blood flow.^11,30^ For more rapid changes in BP, cerebral blood flow exhibits greater variability, in line with the present study; the buffer capabilities in changes in BP over seconds to minutes is referred to as dynamic autoregulation.^11^ Methods for determining individual lower limits of autoregulation and for quantifying autoregulation have been suggested but are less studied in children.^20,25,26,31,32^ During surgery, anesthetic drugs, vasoactive medications, CPB with hypothermia among other factors can also influence autoregulatory capabilities.^33,34^ PaCO2 is a strong influencer of cerebral perfusion pressure.^35^ We monitored EtCO2 continuously during mechanical ventilation, but during CPB blood gasses were only reported at selected timepoints. We did not, however, see large fluctuations in these parameters.

Real-time monitoring of CBFV could potentially enable timely intervention to prevent or mitigate cerebral damage. Mispositioning of cannulas and occlusions can be detected and corrected without delay. This material shows data from different surgical periods that allow for comparison of selected stable phases of surgery. However, looking only at predefined timepoints will exclude clinically relevant rapid changes and events in between phases that continuous monitoring could provide. We explored different hemodynamic parameters that could provide a more individual assessment and management of cerebral hemodynamics in vulnerable patients during complex surgery. Continuous monitoring of CBFV with TCD has been shown to be fast and accurate also in children undergoing cardiac surgery but is limited by a learning curve, experience in operators and poorly suited fastening mechanisms for neonates.^6^ A transfontanellar approach with serial evaluations has been shown to be feasible but does not provide the benefits of continuous monitoring.^9,10^

Different monitoring and treatment strategies to prevent cerebral damage are used during cardiac surgery. Hypothermia is a key strategy to reduce cerebral ischemic events by lowering the brain’s metabolic demands. Deep hypothermia with circulatory arrest and selective antegrade cerebral circulation are alternative strategies in high-risk patients but are both associated with white matter injuries.^36^ Arterial pressure, venous pressure, CPB arterial line pressure, pulse oximetry, blood gases, venous saturation are commonly used in monitoring these children.^37^ Current cerebral monitoring strategies include electroencephalography (EEG), cerebral oximetry with NIRS and TCD. EEG can be used evaluate perioperative electrical activity to assess the effect of anesthesia and hypothermia, detect seizures and direct cerebral protection. However, there is lack of data demonstrating benefits and it is not routinely used.^38^ NIRS is more used in the perioperative clinical setting.^37^ NIRS is noninvasive, easy to apply and provides real time information on regional cerebral oxygenation. However, detection of hyperperfusion is challenging with NIRS as rScO2 of 95% is the upper limit and a hyperperfused brain can have a stable rScO2 of 95%. In this study we also demonstrate the limitation of NIRS in an example with accidental occlusion of the arterial cannula where CBFV increased threefold from baseline after reperfusion, while rScO2 remained at 95%. The relationship between transfontanellar cerebral Doppler and NIRS measurements, or NIRS estimated cerebral oxygen extraction, is interesting and could provide valuable insights in evaluating cerebral metabolism. In phase 4 of this study, the phase with the lowest measured temperature, the relative decrease in NIRS was lower compared to CBFV which can indicate that the reduction in metabolism is more profound than the reduction in flow. However, due to the heterogenicity of this group this would be better to explore in a more selected group. Multimodal neurophysiological monitoring and intervention algorithms based on EEG, venous oxygen saturation and TCD has been proposed in pediatric patients.^39^ For all technologies the correlation between monitoring findings and clinical outcome is still unknown.

### Limitations

The main limitation of this pilot study is the relatively small sample size and the heterogeneity of diagnosis and procedures performed. However, the amount of monitoring data including the total number of Doppler measurements in each patient is very high. Furthermore, we have not correlated our results to clinical outcomes or magnetic resonance imaging.

Mxa as a single parameter to evaluate autoregulation is a simplification of the complex cerebral autoregulation process. Autoregulation is influenced by numerous factors beyond the scope of this pilot study, including pressure, flow resistance, temperature, rate of temperature change, hematocrit levels and undesired vasoplegia.

The NeoDoppler system relies on acquiring adequate signal quality evaluated by color M-mode and the Doppler-spectrum quality through the fontanelle. The exact blood vessels monitored are not truly known but are most often the arteria cerebri anterior and branches. A shift in probe position can potentially cause change of the angle of insonation and cause a slight change in velocity measurements. To follow a velocity trend over time the probe should be kept in the same position. A robust and gentle attachment mechanism is key, especially in awake children.

### Future perspectives

After this study the probe attachment was improved to make it even more robust yet still gentle to avoid probe movement during monitoring. We would also like to study the effects of vasoactive medications and inotropes on CBFV.

In future research, we plan to monitor selected high-risk neonates with transfontanellar Doppler in the post-natal transitional and perioperative period to gain further cerebrovascular insights during these important periods.

In the future, machine learning and AI can be important in gaining more insight into the large amount of cerebral hemodynamic data this technology provides for classifications of patterns, detection of abnormalities and potentially predictive modeling.

## Conclusion

In this study we found that continuous transfontanellar cerebral Doppler monitoring is feasible during cardiac surgery in infants with an open fontanelle. It can provide continuous information on CBFV trends and events. We found a high proportion of time with impaired cerebral autoregulation based on Mxa compared to previous studies. Transfontanellar cerebral Doppler monitoring could become a useful tool to improve cerebral monitoring, to guide anesthesiologists, surgeons and perfusionists during cardiac surgeries with CPB. NIRS and continuous cerebral Doppler together, can potentially provide new pathophysiological insight in these children, but further studies are needed to study possible outcome of more extensive monitoring.

## Supplementary information

Supplementary Information

Supplementary Video1

Supplementary Video2

## Data Availability

The datasets generated during and/or analyzed during the current study are available from the corresponding author on reasonable request.
