# Supplementary material for: Cerebral blood flow dynamics during cardiac surgery in infants
Source: Pediatr Res. 2024 Apr 3;97(2):625–33. doi: 10.1038/s41390-024-03161-z (PMC12014472; doi:10.1038/s41390-024-03161-z)

Supplementary table 1. Technical specifications.

Technical specifications	
Probe frequency (MHz)	7.81
Frame rate (Hz)	424
Imaging diameter (mm)	10.0
Sample volume (mm)	5.00
Imaging depth (mm)	21.7 ± 5 .77
Mechanical index	0.062
Thermal index soft tissue	0.587
Thermal index cranial bone	0.409

Technical specifications of the cerebral Doppler monitor.

Supplementary table 2. Anesthetics.

	N (%)
Sevoflurane induction	11 (73%)
Ketamine induction	3 (20%)
Propofol induction	1 (%)
Isoflurane maintenance	15 (100%)
Thiopental	13 (87%)
Fentanyl	15 (100%)
Midazolam	13 (87%)
Cisatracurium	15 (100%)

Anesthetics used during surgery.

Supplementary Video 1. Transitioning to cardiopulmonary bypass (CPB).

This video demonstrates a Doppler spectrogram with cerebral blood flow velocities in a 6-month-old patient with complete AVSD transitioning to CPB. The video shows a pulsatile flow pattern before CPB transitioning to a more continuous flow pattern when the patient is on CPB. Time averaged velocity (TAV) and resistive index (RI) is periodically displayed.

CPB = cardiopulmonary bypass, TAV = time averaged velocity

Thumbnail Supplementary Video 1:

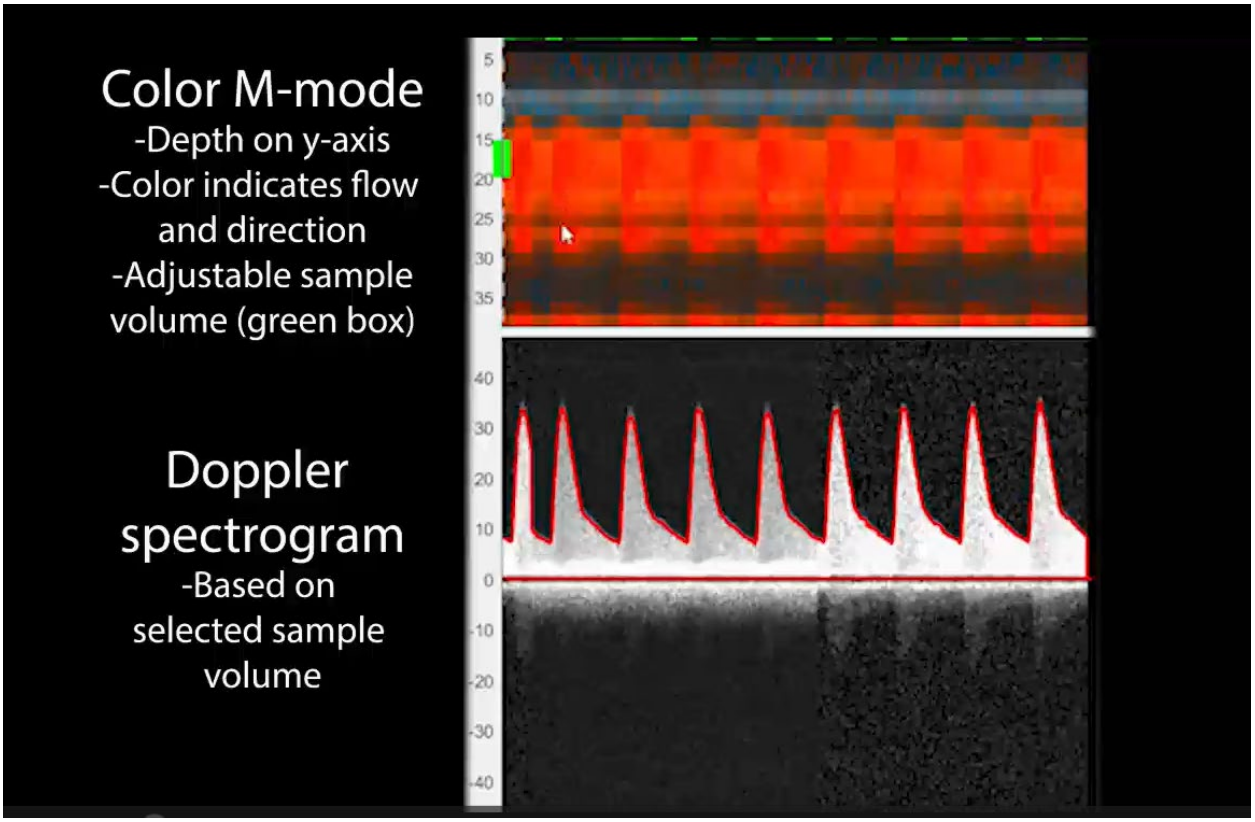

Supplementary Video 2. Occlusion of the arterial cannula.

This video shows the cerebral blood flow velocities during occlusion and reperfusion in a case of accidental occlusion of the arterial cannula during selective perfusion. The moment of occlusion and reperfusion is clearly seen in the Doppler signal. The video also demonstrates the increase in cerebral blood flow velocities after reperfusion.

Thumbnail Supplementary Video 2:

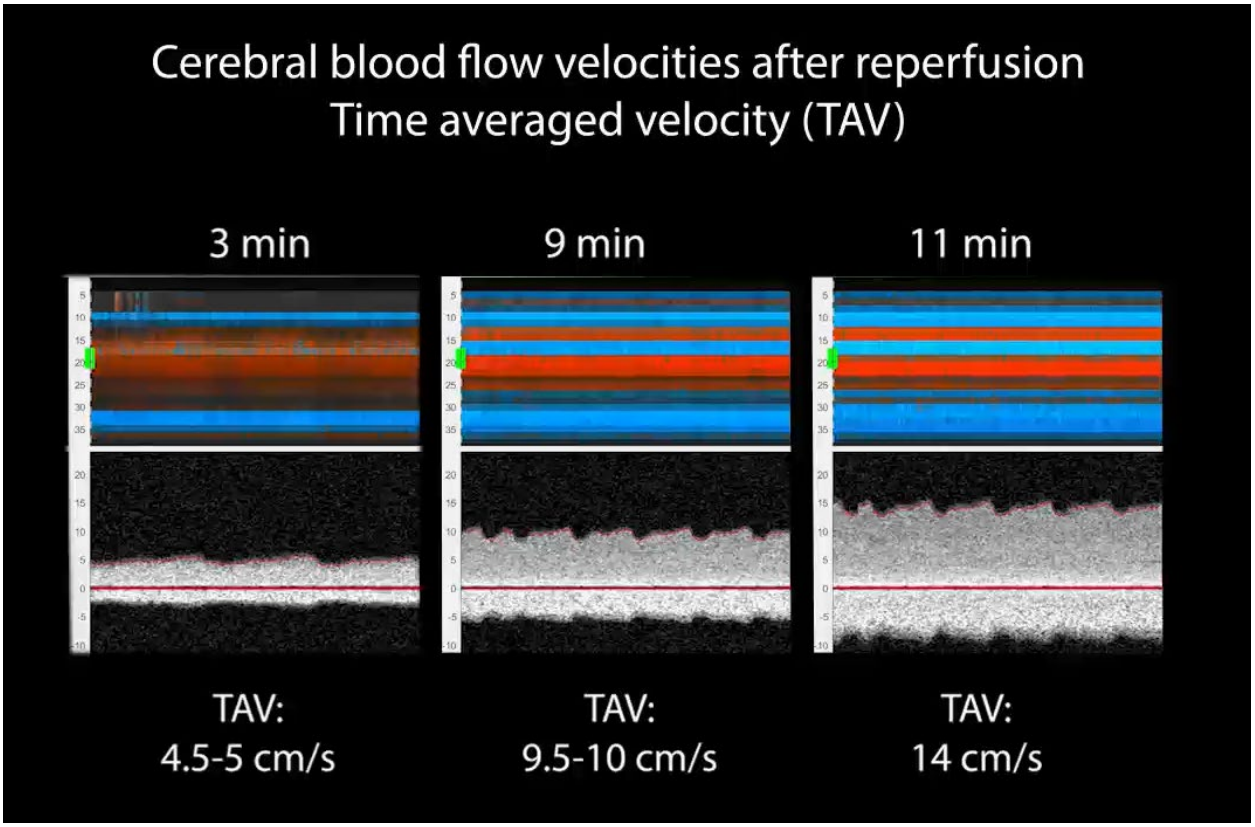

Supplement: Supplementary file 1 — Supplementary Information [file 41390_2024_3161_MOESM1_ESM.pdf]
